# Novel Technology Support Program for Older Adult Program With Interprofessional Geriatrics Students

**DOI:** 10.1093/geroni/igab046.2400

**Published:** 2021-12-17

**Authors:** Bonnie Olsen, Freddi Segal-Gidan, Erin Thayer, Yeini Guardia, M Christina Penate, Alexis Coulourides Kogan

**Affiliations:** 1 University of Southern California - Keck School of Medicine, Alhambra, California, United States; 2 USC Keck School Of Medicine, Los Angeles, California, United States

## Abstract

During the COVID-19 pandemic, many older adults were not receiving primary care services because they could not negotiate the technology for telehealth visits. Coupled with persisting pandemic physical distancing, increased social isolation in older adults was- and continues to be a significant problem. To combat these issues, we aimed to 1) prepare older adults for longitudinal isolation by encouraging social connectedness, and 2) enable older adults to safely access remote primary care services during the pandemic. We paired older adults from 9 housing sites in Los Angeles, CA with health professions graduate students from 9 programs at USC (N = 88 dyads) and provided iPhones to participants without a smartphone. Students educated and supported older adults about the use of technology to access primary care services and to socially connect with family/friends. When requested, 3 additional students provided enhanced 1:1 technology support. Among the 45 participating older adults who received iPhones (51.1%), 22 requests were made for enhanced technology assistance during the 6-month program. Most requests related to initial setup/navigation of iPhone (81.8%) or video calls (27.3%), where others requested help with Wi-Fi (13.6%), composing emails (4.5%), and adding language/translation features (4.5%). Nineteen (83%) technology support requests were successfully resolved; the remaining were unresolved due to loss to follow-up. Our findings demonstrate that older adults can successfully cross the digital divide when technology support is provided. Additionally, pairing older adults with health professions students is an effective strategy to enable access to remote primary care and social connectedness.

